# The association between 24‐hr movement patterns and self‐harm incidence in adolescence

**DOI:** 10.1111/jcpp.70199

**Published:** 2026-07-09

**Authors:** Siyi Huang, Jiameng Cui, Qida He, Shahmir H. Ali, Qi Wang, G. Neil Thomas, Linyan Li

**Affiliations:** ^1^ Department of Infectious Diseases and Public Health City University of Hong Kong Hong Kong China; ^2^ Department of Sports Science, College of Education Zhejiang University Hangzhou China; ^3^ Department of Big Data in Health Science, School of Public Health Zhejiang University School of Medicine Hangzhou China; ^4^ Saw Swee Hock School of Public Health National University of Singapore Singapore Singapore; ^5^ Department of Data Science City University of Hong Kong Hong Kong China; ^6^ Department of Applied Health Sciences, College of Medicine and Health University of Birmingham Birmingham UK

**Keywords:** Non‐suicidal self‐harm, youth mental health, light‐intensity physical activity, compositional data analysis, Millennium Cohort Study

## Abstract

**Background:**

Self‐harm is a pressing public health issue, particularly among adolescents. Physical activity is associated with reduced self‐harm among youths, but evidence is limited. Most studies rely on self‐reported measures and are primarily focused on moderate‐to‐vigorous physical activity (MVPA), often overlooking the role of light‐intensity physical activity (LIPA). To address these gaps, we investigated the prospective associations between accelerometer‐measured 24‐hr movements and subsequent self‐harm in adolescents.

**Methods:**

This prospective cohort investigation analysed 2,016 children from the UK Millennium Cohort Study using data from the age 7 years survey (MCS4) when accelerometers were introduced (mean age = 7.23 years, SD = 0.25). Accelerometer‐measured duration of MVPA, LIPA, sedentary behaviour (SB), and sleep were analysed. Self‐harm behaviours were assessed via validated self‐report questionnaires at ages 14 and 17 years, and subsequently, the associations examined using logistic and compositional data models.

**Results:**

Among 2,016 participants (48.6% female), 548 self‐harm cases occurred during 18,696 person‐years of follow‐up, with a median follow‐up duration of 8.5 years (cumulative incidence = 27.2%). LIPA was inversely associated with self‐harm (OR 0.76, 95% CI: 0.65–0.88). This association was stronger in compositional models (OR 0.23, 95% CI: 0.11–0.47). MVPA showed a significant non‐linear, inverted U‐shaped association with self‐harm (*p* for non‐linearity = .02). The risk peaked at approximately 1.2 hr/day and decreased substantially at levels exceeding 1.7 hr/day, while SB and sleep were not significantly associated with self‐harm. Stratified analyses confirmed robustness.

**Conclusions:**

Our findings reveal a pronounced inverse association between LIPA and self‐harm, along with a non‐linear association for MVPA. These results support 24‐hr movement guidelines that emphasise activity transitions over isolated targets and highlight LIPA's role in early prevention strategies.

## Introduction

Adolescent self‐harm has become an escalating global mental health crisis, presenting a formidable challenge to clinicians worldwide (Zhao et al., 2024). While it occurs across all age groups, adolescents are particularly vulnerable, with lifetime prevalence reaching 14% in adolescents, significantly exceeding the rates typically observed in adult populations (approximately 3% annually; Hawton, Saunders, & O'Connor, 2012). According to the World Health Organisation (WHO), self‐inflicted injuries are the second leading cause of mortality among girls aged 15–19 years globally (Masquelier et al., 2021). Adolescents who engage in self‐harm behaviours face a significantly increased risk of suicide and are nearly 19 times more likely to die by suicide than their peers (Blanchard, Chihuri, DiGuiseppi, & Li, 2021). These trends underscore the importance of identifying modifiable risk factors for self‐harm and developing early preventive strategies in youth.

Research has indicated that self‐harm is influenced by a complex interplay of genetic predispositions, environmental exposures, and modifiable lifestyle factors (Maciejewski et al., 2014; Sokolowski, Wasserman, & Wasserman, 2020). Among the latter, daily movement patterns represent one of the most accessible and modifiable domains. Daily movements encompass moderate‐to‐vigorous physical activity (MVPA; e.g. running, cycling), light‐intensity physical activity (LIPA; e.g. slow walking), sedentary behaviours (SB; e.g. screen time, prolonged sitting), and sleep duration. The evidence indicates that regular physical activity can reduce the risk of self‐harm and even help prevent its most severe outcomes (such as suicide attempts) in adults (Jarvi, Hearon, Batejan, Gironde, & Björgvinsson, 2017; Jung, Lee, Lee, & Lee, 2025; Laflamme, Vaez, Lundin, & Sengoelge, 2022). However, evidence for such a protective association in adolescents remains limited and inconclusive. Following a systematic review of available literature, only a limited number of studies have examined the association between physical activity and self‐harm (Gao, Lu, Zhang, Han, & Hu, 2024; Lai et al., 2021; Liu, Zhang, Kamper‐DeMarco, Hu, & Yao, 2020; O'Reilly et al., 2022; Yu, Mu, & Li, 2023). Among these, most studies focused on structured exercise programmes and MVPA, and the findings remained inconsistent (Gao et al., 2024; Lai et al., 2021; Liu et al., 2020; O'Reilly et al., 2022; Yu et al., 2023). Several investigations have reported an inverse association between physical activity levels and the incidence of self‐harm, but a recent large‐scale study involving 13,349 Chinese high school students found no statistically significant association between the frequency of MVPA and self‐harm behaviours (Liu et al., 2020).

Currently, most existing studies examining the association between physical activity and adolescent self‐harm rely on self‐reported data. While self‐reports are cost‐effective and easy to administer, their reliability and validity are more limited. LIPA (Füzéki, Engeroff, & Banzer, 2017), in particular, often occurs in brief, sporadic episodes throughout the day, making it more challenging for individuals to accurately recall the duration and intensity of their movements compared with MVPA (Felez‐Nobrega et al., 2021; Füzéki et al., 2017). Consequently, the health effects of LIPA remain uncertain. Objective measurement tools, such as accelerometers, offer a valuable alternative by providing continuous and reliable data on low‐intensity movement and enabling more precise evaluation of the association between LIPA and mental health outcomes (Felez‐Nobrega et al., 2021; Füzéki et al., 2017). Additionally, previous investigations on the link between physical activity and self‐harm have been mostly cross‐sectional, limiting the ability to establish temporal sequence and clarify the directionality of the associations (Felez‐Nobrega et al., 2021; Füzéki et al., 2017). This is important as psychological outcomes often develop over a longer period than typically recognised.

Notably, middle childhood (approximately ages 6–12 years) represents a critical developmental period for shaping mental health trajectories and establishing behavioural patterns (Boyden, Dawes, Dornan, & Tredoux, 2019; Knafo‐Noam, Daniel, & Benish‐Weisman, 2024). During this phase, the transition to formal schooling reinforces behavioural regularity (Dunn, Slomkowski, & Beardsall, 1994), while the primary drivers of social influence shift from the immediate family to peers (Pedersen, Vitaro, Barker, & Borge, 2007). Within this stabilising environment, systematic reviews indicate that physical activity behaviours exhibit moderate stability, or ‘tracking’, from early childhood through adolescence (Hayes, Dowd, MacDonncha, & Donnelly, 2019; Telama et al., 2014). Beyond behavioural continuity, this stage constitutes a sensitive window for neurobiological and psychological maturation. Consequently, cultivating positive lifestyle behaviours during middle childhood can mitigate early vulnerabilities and foster healthy developmental trajectories that persist into adolescence (Jones, Schinka, van Dulmen, Bossarte, & Swahn, 2011).

To address these research gaps, the primary objective of this study was to investigate the prospective associations between accelerometer‐measured 24‐hr movement patterns and the risk of self‐harm behaviours during adolescence using data from the Millennium Cohort Study. This study integrated accelerometer‐measured 24‐hr movement behaviours with a compositional analytical framework to quantify the interdependent associations between different 24‐hr movement patterns and their collective association with adolescent self‐harm. These findings have the potential to directly inform school‐based, community, and clinical interventions, as well as public health strategies that promote balanced daily movement patterns as a means of preventing self‐harm in adolescents.

## Methods

### Study design and population

We used data from the UK Millennium Cohort Study (MCS), a nationally representative longitudinal cohort target population of children born in the United Kingdom between 1 September 2000, and 11 January 2002. Initial data collection began when participants were 9 months old, with follow‐up assessments at ages 3 (MCS2), 5 (MCS3), 7 (MCS4), 11 (MCS5), 14 (MCS6), and 17 (MCS7) years (Connelly & Platt, 2014). Stratified random sampling was used to ensure demographic representation, with an intentional oversampling of children from socioeconomically disadvantaged and ethnically diverse backgrounds. Further methodological details are publicly available via the MCS official website (https://cls.ucl.ac.uk/cls‐studies/millennium‐cohort‐study/).

Although the MCS cohort was recruited at birth, the current analysis considers wave 4 (Age 7 years) as the baseline time point, as this was the first wave to include objective physical activity measurement. The study included individuals with valid accelerometer data collected at this wave (MCS4) and responses to self‐harm questions from the Youth Self‐completion Questionnaire at the MCS6 and MCS7 follow‐ups (Ougrin & Boege, 2013). A flowchart detailing the inclusion and exclusion criteria for the study participants is provided in Figure S1.

### Assessment of the 24‐hr time‐use composition

At age 7 years (MCS4), 24‐hr movement behaviours were measured using Actigraph GT1M accelerometers worn for 7 days. Raw accelerometer data were processed to derive time spent in MVPA, LIPA, SB, and sleep. Detailed information on wear‐time criteria, classification of activities using counts per minute, and handling of non‐wear periods (e.g. swimming, sleep) is provided in Figure S1 and the MCS technical reports (Butte et al., 2018; Evenson, Catellier, Gill, Ondrak, & McMurray, 2008; Geraci, Rich, Sera, Cortina‐Borja, & Griffiths, 2012; Love, Adams, Atkin, & van Sluijs, 2019; Pulsford et al., 2011).

According to this same technical report (Geraci et al., 2012), the Institute of Child Health (ICH) classifies activity intensity using counts per minute (cpm) as follows: sedentary (<100 cpm), light (100–2,240 cpm), moderate (2241–3,840 cpm), and vigorous (≥3,841 cpm) (Evenson et al., 2008; Love et al., 2019; Pulsford et al., 2011). Time spent swimming and cycling, as recorded by guardians, was also included in the measurement of MVPA. Periods when the device was not worn, excluding the recorded times for swimming and cycling, were considered sleep. To ensure reliability in activity estimates, included participants were required to have at least 3 days of valid accelerometer data, including at least one weekend day and 2 weekdays, with a minimum wear time of 10 h during waking hours each day (Love et al., 2019).

### Measurement of self‐harm behaviours

Data on self‐harming behaviours were systematically collected using a simplified version of the self‐harm inventory (SHI) through the youth self‐completion questionnaire (Geraci et al., 2012). In MCS6, adolescents were asked, ‘In the past year, have you hurt yourself on purpose in any way?’ In the subsequent wave (MCS7), participants were questioned about specific behaviours in the previous year, including: ‘(1) Cut or stabbed yourself, (2) Burned yourself, (3) Bruised or pinched yourself, (4) Taken an overdose of tablets, (5) Pulled out your hair, (6) Hurt yourself in some other way’. Each item was provided with a dichotomous response option: ‘yes’ or ‘no’. A participant was classified as a self‐harm case if they responded affirmatively to any of these behaviours during either the MCS6 or MCS7 assessment periods (Yang, Jia, Lu, & Jia, 2023). Outcomes were collapsed across these two time points to address the relatively low base rates of self‐harm at individual waves, thereby ensuring sufficient statistical power for the compositional data analysis (CODA).

### Covariates

The selection of covariates was informed by existing literature and guided by a directed acyclic graph (DAG) to visually map hypothesised associations between variables. Following methodological recommendations for applied health research (Tennant et al., 2021), the DAG was utilised specifically to identify a minimal sufficient adjustment set of observed confounders. This approach ensured that the multivariable models were adjusted for relevant covariates while avoiding over‐adjustment (e.g. adjusting for mediators). Crucially, as depicted in the DAG, we acknowledged the presence of unmeasured confounders (e.g. genetic factors) that could not be adjusted for.

The following covariates were included due to their associations with both the exposures and the outcome of interest: age, sex (male or female), ethnicity (White or Other race), annual family income, parental marital status, BMI *z*‐score (Body Mass Index *z*‐score), child's history of any mental disorders (parent‐reported at age 7 years), parents' own history of mental disorders, interpersonal difficulties (parents reported whether their child's difficulties interfered with their friendships), and residence (urban/town and fringe/village, hamlet, and isolated dwellings). According to the DAG, these variables were selected based on their established associations with self‐harm and daily movement patterns, ensuring a rigorous and well‐specified model. Further methodological details are provided in Appendices S1–S6, with the DAG‐based selection of covariates described in Appendix S6.

Participant's sex was recorded at birth (or reported by parents) in the MCS baseline survey. Race and ethnicity were ascertained through parental self‐report, based on the categories used in the UK national census. Data on gender identity were not collected in the MCS due to the study's original design and timeline.

### Equity, diversity and inclusion statement

This study utilised a nationally representative cohort oversampled for children from socioeconomically disadvantaged and ethnically diverse backgrounds. However, our analyses were limited to variables available in the public dataset and did not explicitly stratify results by ethnicity or income due to power concerns.

### Patient and public involvement

Patients and the public were not involved in the design, conduct, reporting, or dissemination plans of this research.

### Ethical statement

This study received ethical approval from the UK Millennium Cohort Study Law and Ethics Committee and local research ethics boards. Written informed consent was obtained from parents at each wave, and additional written assent was secured from the adolescents themselves at ages 14 and 17 years after study procedures were fully explained. The current study methods and use of de‐identified data were further reviewed and ethically approved by the Medical Ethics Committee of the Department of Psychological and Behavioural Sciences at Zhejiang University (Approval No. 2022060).

### Statistical analysis

Given that this was a secondary analysis of the Millennium Cohort Study (MCS), the sample size was fixed by data availability. However, the final analytic sample of 2,016 participants (including 548 self‐harm cases) was considered sufficient to provide robust estimates for the logistic regression models. Continuous variables were reported as means with standard deviations (SD), whereas categorical variables were summarised using frequencies and percentages. In the descriptive analyses, compositional means and log‐ratio differences were used to compare MVPA, LIPA, SB, and sleep between individuals with and without self‐harm behaviours. A variation matrix was utilised to illustrate the associations and co‐dependencies among these daily movement behaviours (i.e. MVPA, LIPA, SB, and sleep) (Aitchison, 1982; Chastin, Palarea‐Albaladejo, Dontje, & Skelton, 2015). In CODA, the variation matrix quantifies the log‐ratio variances between pairs of components, helping identify which behaviours tend to substitute or co‐occur with one another (Appendices S1 and S2; Tables S1 and S2).

For the association analysis, we employed logistic regression models for single behaviours and CODA (Greenacre, 2021) to assess the association between movement behaviours and self‐harm. This approach is used to understand how different co‐dependent behaviours (such as physical activity, SB, and sleep) are proportionally distributed within a fixed time frame (like 24 hours)(McGregor et al., 2020). To account for the co‐dependence of behaviours, the CODA models utilised an isometric log‐ratio (ILR) transformation (Hron et al., 2012). After the transformation, the sum of the transformed values for the four movements (MVPA, LIPA, SB, and sleep) equals one. Both model types were adjusted for age, sex, ethnicity, BMI *z*‐score, residence, annual family income, child and parent diagnosed mental health disorders, interpersonal difficulties, and parental marital status. Model outcomes were presented using ternary heatmaps, and we used restricted cubic spline (RCS) analyses to investigate potential non‐linear relationships.

Recent studies have demonstrated that the association between physical activity and mental health is often complex and non‐linear, characterised by threshold effects, plateaus, or curvilinear trajectories (Chen, Xin, Jia, Tu, & Li, 2025; Zhang et al., 2025). Relying solely on linear models might obscure these nuanced associations. Consequently, we adopted the RCS approach, strictly adhering to the methodological guidelines by Desquilbet and Mariotti (2010). Sensitivity analyses were performed examining self‐harm at ages 14 and 17 years separately. The corresponding supplementary estimates are presented in Tables S3 and S4. To assess the missing data mechanism, we first performed Little's Missing Completely at Random (MCAR) test, which yielded a significant result (χ^2^ = 2,941, *p* < .001), indicating that the data were not MCAR. Consequently, missing data were handled using multiple imputation (MICE package; van Buuren & Groothuis‐Oudshoorn, 2011) under the missing at random (MAR) assumption. The plausibility of this assumption was supported by the analysis of missingness (Table S5), which revealed that missing data were significantly associated with observed characteristics at age 7 years, including sex, ethnicity, BMI, family income, and parents' marital status. Accordingly, the imputation model was specified to include the outcome variable (self‐harm), all exposure variables, and these significant predictors of missingness are identified in Table S5.

Detailed explanations of the compositional mean, variation matrix, log‐ratio difference, CODA/ILR transformation, ternary plots, and confounder selection are provided in Appendices S1–S6.

All statistical analyses were conducted in R (version 4.4.2) with a two‐tailed *p* value <.05 considered significant.

## Results

This study included 2,016 participants, comprising 1,035 boys and 981 girls, with a mean age of 7.23 years (SD = 0.25) at baseline. The recruitment flow and sequential sample selection process are illustrated in Figure S1. Starting from the initial MCS cohort, the final analytical sample of 2,016 participants was reached after exclusions primarily attributable to invalid accelerometer data or loss to follow‐up. Most participants (86.51%) were identified as White and resided in urban areas (61.81%). A total of 548 (27.18%) participants reported self‐harm behaviours at ages 14 or 17 years of age (Table 1). As given in Table 1, individuals who reported self‐harm behaviours were more likely to be female, White, experience difficulties maintaining friendships, engage in lower levels of LIPA and MVPA, sleep for longer durations, and have parents with a diagnosed current mental disorder.

**Table 1:** Characteristics of participants at age 7 years by self‐harm (*n* = 2016)

	Overall (*N* = 2016)	Non‐self‐harm (*N* = 1,468)	Self‐harm (*N* = 548)	*p* values^a^
Age (mean (SD))	7.23 (0.25)	7.23 (0.24)	7.23 (0.25)	.99
Sex, *n* (%)				
Boy	1,035 (51.34)	834 (56.81)	201 (36.68)	**<.001**
Girl	981 (48.66)	634 (43.19)	347 (63.32)	
Ethnicity, *n* (%)				
White	1744 (86.51)	1,246 (84.88)	498 (90.88)	.**001**
Others^b^	272 (13.49)	222 (15.12)	50 (9.12)	
Residence, *n* (%)				
Urban	1,246 (61.81)	918 (62.53)	328 (59.85)	.23
Town and fringe	163 (8.09)	108 (7.36)	55 (10.04)	
Village, hamlet, and isolated dwellings	171 (8.48)	122 (8.31)	49 (8.94)	
Missing	436 (21.63)	320 (21.8)	116 (21.17)	
BMI z‐score (mean (SD))	0.56 (1.29)	0.55 (1.30)	0.59 (1.28)	.51
Annual family income (mean (SD))	446.83 (232.36)	443.22 (229.37)	456.51 (240.15)	.26
Children's mental health history, *n* (%)				
Ever/currently	2 (0.10)	1 (0.10)	1 (0.20)	.73
Never	1951 (96.78)	1,420 (96.73)	531 (96.90)	
Missing	63 (3.12)	47 (3.17)	16 (2.90)	
Children's interpersonal difficulties, *n* (%)				
Yes	486 (24.11)	335 (22.82)	151 (27.56)	.**04**
No	1,498 (74.31)	1,106 (75.34)	392 (71.53)	
Missing	32 (1.58)	27 (1.84)	5 (0.91)	
Parents' marriage status, *n* (%)				
Legally Separated^c^	442 (21.92)	312 (21.25)	130 (23.72)	.17
Married	1,536 (76.19)	1,132 (77.11)	404 (73.72)	
Missing	38 (1.88)	24 (1.63)	14 (2.55)	
Parents' mental health history, *n* (%)				
Ever/Currently	181 (8.98)	126 (8.58)	55 (10.04)	.15
Never	1796 (89.09)	1,318 (89.78)	478 (87.23)	
Missing	39 (1.93)	24 (1.65)	15 (2.73)	
MVPA (hr/day) (mean (SD))	1.20 (0.44)	1.22 (0.45)	1.16 (0.41)	.**01**
SB (hr/day) (mean (SD))	6.71 (1.25)	6.70 (1.27)	6.75 (1.20)	.48
LIPA (hr/day) (mean (SD))	4.74 (0.68)	4.78 (0.68)	4.65 (0.68)	**<.001**
Sleep (hr/day) (mean (SD))	11.34 (1.14)	11.30 (1.16)	11.44 (1.10)	.**02**
Wear time^d^ (hr/day) (mean (SD))	12.66 (1.14)	12.69 (1.16)	12.56 (1.10)	.**02**

Bold values indicate statistically significant results at *p* < .05. BMI *z*‐score, body mass index (BMI) *z*‐score; LIPA, Light‐intensity physical activity; MVPA, moderate‐vigorous physical activity; SB, sedentary behaviours; SD, standard deviation.

^a^ Continuous variables were compared using analysis of variance, while categorical variables were compared using Chi‐square tests.

^b^ Represented individuals of Indian, Pakistani, Bangladeshi, or other ethnic backgrounds.

^c^ Indicated Legally Separated/Single/Divorced/Widowed.

^d^ Indicated the average daily wear time of the accelerometer.

Compared with participants who did not report self‐harm, individuals with self‐harm behaviours spent 4.3% less time in MVPA, 2.7% less time in LIPA, 0.9% more time in SB, and 1.1% more time sleeping (Figure 1, Table S1). As shown in the variation matrix (Table S2), the variance of log (LIPA/SB) and log (LIPA/sleep) approached zero, reflecting the highest co‐dependence between these two behaviours.

**Figure 1:**
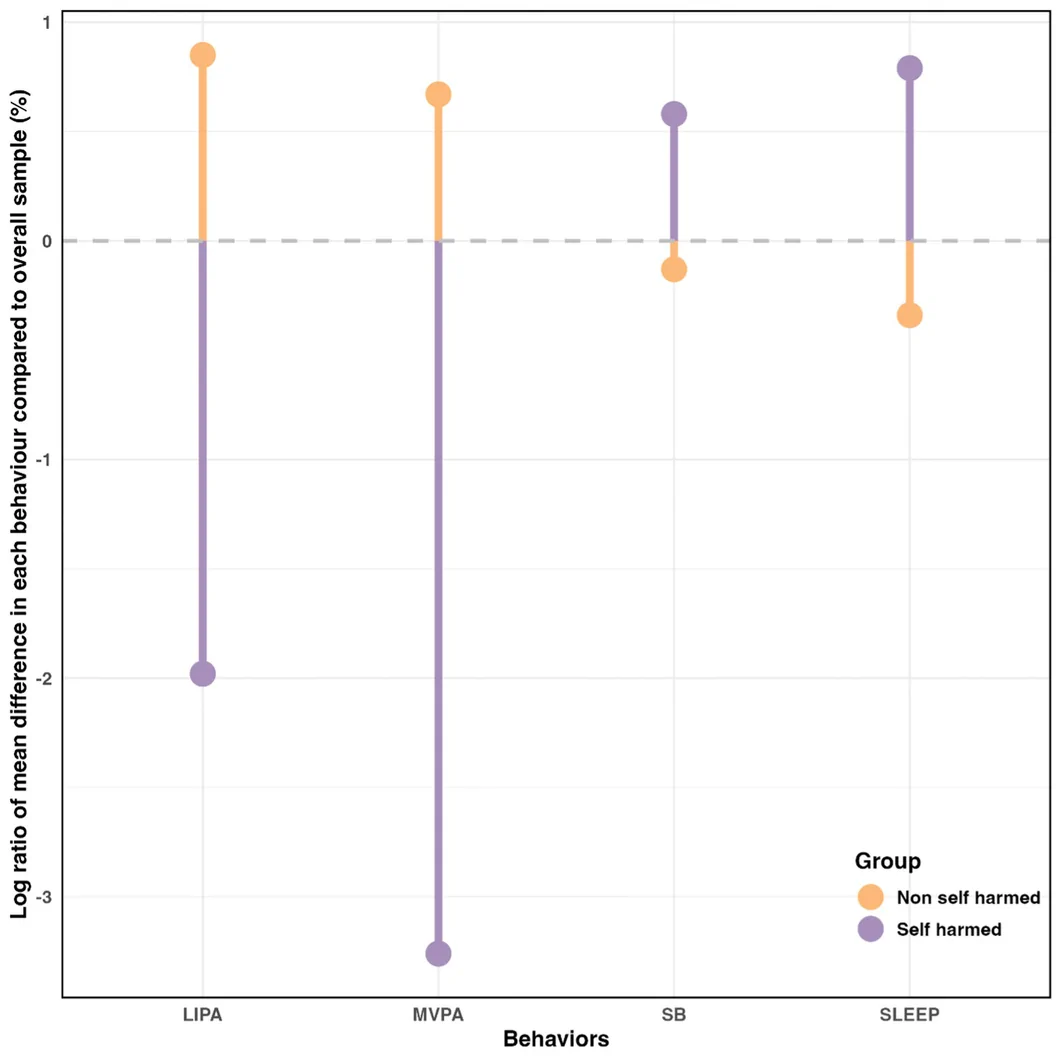
Difference in log‐ratio time spent in each behaviour by self‐harm status compared with the sample mean. The plot illustrates the log‐ratio difference of the mean time spent in four behaviours (LIPA, MVPA, SB, and sleep) between adolescents with self‐harm (orange) and those without (purple), relative to the overall sample's compositional mean. A positive value on the *y*‐axis indicates more time spent in a specific behaviour compared with the average, while a negative value indicates less time. LIPA, light‐intensity physical activity; MVPA, moderate‐to‐vigorous physical activity; SB, sedentary behaviour

During 18,696 person‐years of follow‐up, the incidence rate of self‐harm behaviours was 2.93 per 100 person‐years. In single predictor models, LIPA was associated with a lower risk of self‐harm behaviours (odds ratio [OR] = 0.76, 95% CI: 0.65–0.88, *p* < .0001), after adjusting for sociodemographic factors and mental health conditions (Table 2). In RCS analysis, a significant non‐linear, inverted U‐shaped association between MVPA and self‐harm behaviours was observed (*p* for non‐linearity = .02). The estimated risk increased initially, peaking at approximately 1.2 hr/day, before declining to imply a potential protective effect at durations exceeding 1.7 hr/day (Figure 2I). No significant associations were observed between SB or sleep and self‐harm behaviours.

**Table 2:** Single predictor and compositional data analysis models (CODA) of how daily time spent on activity affects self‐harm for each behaviour

Behaviours	Single predictor^a^		CODA^b^	
	OR (95% CI)	*p‐*values	OR (95% CI)	*p*‐values
MVPA	0.97 (0.75–1.23)	.78	1.08 (0.79–1.47)	.63
LIPA	0.76 (0.65–0.88)	**<.0001**	0.23 (0.11–0.47)	**<.0001**
Sleep	1.04 (0.95–1.15)	.39	1.20 (0.67–2.17)	.55
SB	1.06 (0.97–1.15)	.19	1.29 (0.86–1.93)	.22

MVPA, Moderate‐vigorous physical activity; LIPA, Light‐intensity physical activity; SB, sedentary behaviour.

Single predictor and CODA models adjusted for age, sex, ethnicity, body mass index (BMI) *z*‐score, residence, family income, children's mental disorder, children's interpersonal difficulties, parents' mental disorder, and parents' marriage status.

^a^ Single predictor model: The exposure variable was used to assess the univariate association between each behaviour and the outcome by evaluating the time allocated to a single behaviour.

^b^ CODA model: The regression coefficient reflected the variation in the log‐ratio of a specific behaviour relative to the others.

**Figure 2:**
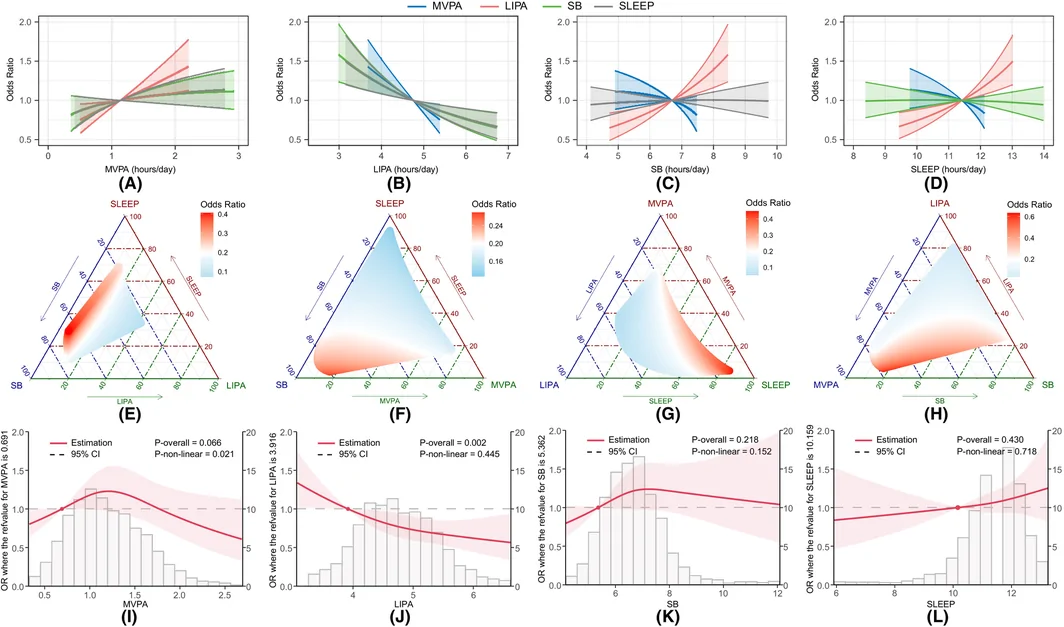
Associations between 24‐hr movement compositions and self‐harm risk. This figure integrates three different visual representations of the association between time‐use compositions of physical activity, sedentary behaviour, and sleep with self‐harm risk. All analyses were adjusted for age, sex, ethnicity, BMI *z*‐score, residence, family income, children's mental disorders, interpersonal difficulties, parental mental health, and parents' marital status. Alternative Compositions and Self‐Harm Risk (Upper Left Panel, A–D): Explores the impact of reallocating time to or from one behaviour relative to the others on self‐harm risk. The intersection point of the lines represents the sample compositional mean, which serves as the reference: 68 min (1.14 hr) of MVPA, 285 min (4.75 hr) of LIPA, 402 min (6.69 hr) of SB, and 685 min (11.42 hr) of sleep. Heatmap Ternary Diagrams (Upper Right Panel, E–H): Displays the expected odds ratios for self‐harm derived from Model 2 across different compositions of moderate‐to‐vigorous physical activity (MVPA), light‐intensity physical activity (LIPA), sedentary behaviour (SB), and sleep. In each plot, one behaviour is held constant while the other three vary. The colours represent the odds ratio, with red indicating a higher risk and blue indicating a lower risk. Restricted Cubic Spline Models (Bottom Panel, I–L): Shows the adjusted odds ratios (solid lines) and 95% confidence intervals (shaded areas) for self‐harm risk across different durations of MVPA, LIPA, SB, and sleep. Histograms indicate the distribution of each movement behaviour in the study population

In the CODA models, based on the ILR transformation, LIPA was included while accounting for the influence of the other three behaviours. After adjusting for the remaining movement components, a stronger and more significant association between LIPA and self‐harm behaviours was observed (OR = 0.23, 95% CI: 0.11–0.47, *p* < .001). Subsequently, in the compositional logistic regression, we assessed the association between the optimal set of ILR coordinates Z = (Z1, Z2, Z3) and the risk of self‐harm. Specifically, Z1 represents the ratio of LIPA time in relation to time in MVPA, SB, and sleep; Z2 represents the ratio of MVPA time in relation to time in SB and sleep; and Z3 represents the ratio of SB time in relation to time in sleep. These ILR coordinates were used to construct a model of the effect on the outcome after substituting one behaviour for another while holding the other two behaviours constant through isotemporal analysis. As shown in Table 3, in the compositional logistic regression, the association of Z1 with a reduced risk remained significant (OR = 0.22, 95% CI: 0.11–0.46, *p* < .0001). However, Z2 (OR = 0.91, 95% CI: 0.66–1.26, *p* = .57) and Z3 (OR = 0.95, 95% CI: 0.52–1.78, *p* = .86) showed no significant effects. Likelihood ratio tests revealed significant differences between the more complex model and the baseline model based on covariates (*p* < .0001). The goodness‐of‐fit of the compositional logistic regression model was assessed using the Hosmer–Lemeshow test, which yielded a *p* value of .89.

**Table 3:** Compositional logistic regression model coefficient estimates and 95% confidence interval

	ILR coordinates^a^	OR (95% CI)^b^	*p* values^c^
Model 1	*Z*1	0.25 (0.12–0.49)	**<.0001**
	*Z*2	0.85 (0.62–1.17)	.32
	*Z*3	1.40 (0.78–2.55)	.27
	Overall	–	.**001**
	Hoslem‐test		.80
Model 2	*Z*1	0.22 (0.11–0.46)	**<.0001**
	*Z*2	0.91 (0.66–1.26)	.57
	*Z*3	0.95 (0.52–1.78)	.86
	Overall	–	**<.0001**
	Hoslem‐test		.89

To account for the interactions between the four movement behaviours, we performed an isometric log‐ratio (ILR) transformation on the data before incorporating it into the CODA model. The ILR coordinates were defined as follows: Z1 represents the ratio of LIPA time in relation to time in MVPA, SB, and sleep; Z2 represents the ratio of MVPA time in relation to time in SB and sleep; and Z3 represents the ratio of SB time in relation to time in sleep. Bold values indicate statistically significant results at *p* < .05. LIPA, light‐intensity physical activity; MVPA, moderate‐to‐vigorous physical activity; SB, sedentary behaviour.

^a^ *Z* was defined as: $Z=\left(\sqrt{\frac{3}{4\;}\;}\ln\frac{\text{LIPA}}{{\left(S\cdot \mathrm{SB}\cdot \text{MVPA}\right)}^{1/3}},\sqrt{\frac{2}{3\;}\;}\ln\frac{\text{MVPA}}{{\left(S\cdot \mathrm{SB}\right)}^{1/2}},\sqrt{\frac{1}{2\;}\;}\ln\frac{S}{\mathrm{SB}}\right).$

^b^ OR indicated change in odds ratio per unit increase in the associated ILR coordinates (isometric log‐ratio coordinates). Model 1 adjusted for age and sex. Model 2 additionally adjusted for ethnicity, body mass index (BMI) *z*‐score, residence, family income, parents' mental disorder and parents' marriage status.

^c^ *p* values for individual ILR coordinates were based on Wald tests and overall *p* values were based on likelihood ratio tests (LRT).

In Figure 2A–D, increasing the proportion of LIPA was consistently associated with a reduced risk of self‐harm. Conversely, reallocating time from LIPA to any of the other three movement behaviours significantly increased self‐harm risk. This pattern is clearly demonstrated in Figure 2B, where replacing LIPA with SB, MVPA, or sleep led to a substantial increase in risk. Additionally, Figure 2E–H presents a ternary plot of the predicted odds ratios based on Model 2. In each scenario, one component was held constant, while a range of potential combinations for the remaining three components was explored in relation to the expected probabilities. Figure 2E excludes MVPA, illustrating the risk ratios associated with the sub‐composition (SB, LIPA, sleep) while holding MVPA constant. Figure 2F shows the risk ratios for the sub‐composition (sleep, SB, MVPA) with LIPA held constant. Similarly, Figure 2G presents the risk ratios for the sub‐composition (sleep, LIPA, MVPA) with SB held constant. Finally, Figure 2H depicts the risk ratios for the sub‐composition (MVPA, SB, LIPA) with sleep held constant. Across all scenarios, increasing the proportion of LIPA was associated with a reduced risk of self‐harm. Notably, we observed dose–response associations between the duration of daily movement behaviours (particularly MVPA and LIPA) and self‐harm risk, as shown in the RCS models (Figure 2I–L).

For sensitivity analyses, we conducted separate models to explore the association between daily movement at age 7 years and self‐harm occurring at age 14 years, as well as between daily movement at age 7 years and self‐harm occurring at age 17 years. These sensitivity analyses were conducted to assess the robustness of our findings and to further clarify the temporal relationships. In the single predictor model, LIPA was significantly associated with self‐harm, with OR of 0.81 (95% CI: 0.66–0.99, *p* = .04) in MCS6 and 0.77 (95% CI: 0.65–0.91, *p* = .002) in MCS7. Similarly, in the CODA model, the ORs for LIPA were 0.33 (95% CI: 0.13–0.84, *p* = .02) for MCS6 and 0.29 (95% CI: 0.13–0.63, *p* = .002) for MCS7 (Tables S3 and S4; Figures S2 and S3).

## Discussion

This study is one of the few to simultaneously examine MVPA, LIPA, SB, and sleep in relation to adolescent self‐harm using accelerometer‐based measures within a prospective design. We observed a robust inverse association between LIPA and self‐harm: each additional hour of LIPA per day was associated with a 24% reduction in risk (OR 0.76, 95% CI 0.65–0.88). This association became more pronounced after accounting for the compositional nature of daily time use. Within the compositional model, and relative to the average composition of diurnal activities in our sample, a higher proportion of time spent in LIPA was associated with a 77% lower risk of self‐harm (OR 0.23, 95% CI: 0.11–0.47; *p* < .0001). CODA allowed us to isolate the independent contribution of LIPA while addressing the inherent co‐dependency among movement behaviours. Additionally, an inverted U‐shaped relationship between MVPA and self‐harm behaviours was observed at ages 14 or 17 years (*p* for non‐linearity = .02), suggesting differential effects across activity intensities.

To date, only a limited number of studies have employed objective measures to examine the association between LIPA and adolescent self‐harm, making direct comparisons with our findings challenging. Nonetheless, a broader body of literature supports the association between physical activity and reduced self‐harm risk. Children who establish regular physical activity patterns before adolescence exhibit greater psychological resilience during educational transitions (Dunn et al., 1994; Pedersen et al., 2007). Children engaged in organised sports are more likely to report higher levels of self‐esteem and a lower incidence of self‐harm over time (Latina, Jaf, Alberti, & Tilton‐Weaver, 2022; Lucena, Rossi, Azevedo, & Pereira, 2022; Shan et al., 2024). Notably, adolescents with asthma, rhinitis, or eczema are often considered to be at heightened risk for self‐harm (Medeiros et al., 2025). Even within these vulnerable populations, engagement in physical activity and reduced sedentary time have been associated with a lower risk of self‐harm (Medeiros et al., 2025). Emerging evidence from the UK Biobank's genotype‐specific effectiveness study provides biological support for the mental health benefits of LIPA: appropriate levels of LIPA were linked to a reduced risk of being hospitalised for self‐harm among individuals with depression and a particular genotype (Jung et al., 2025). Collectively, this evidence highlights the potential value of incorporating LIPA promotion into early preventive strategies for self‐harm, particularly among at‐risk children and youth populations.

Building upon these findings, our study highlights the practical and developmental significance of promoting LIPA during early life stages. Our findings underscore the practical and developmental relevance of LIPA in early life. In previous investigations, LIPA has received limited attention as a potential factor in self‐harm prevention compared with MVPA. However, promoting LIPA among children around the age of 7 years may offer a highly pragmatic strategy for reducing future risk of self‐harm, while also being consistent with the broader benefits of light‐intensity activity for physical health and psychological well‐being (Biddle, Ciaccioni, Thomas, & Vergeer, 2019; Felez‐Nobrega et al., 2021; Füzéki et al., 2017). Middle childhood (ages 6–12 years) constitutes a critical developmental window for establishing physical activity and mental health trajectories (Dunn et al., 1994; Pedersen et al., 2007). Systematic reviews indicate that physical activity behaviours exhibit moderate stability, or ‘tracking’, from childhood through adolescence, suggesting that active habits formed during this period create a lasting behavioural foundation (Hayes et al., 2019; Telama et al., 2014). Beyond behavioural continuity, this stage represents a sensitive window for neurobiological and psychological maturation. Physiologically, physical activity promotes neuroplasticity in brain regions essential for emotional regulation, such as the hippocampus and prefrontal cortex (Pagani, Fitzpatrick, Barnett, & Dubow, 2010). Psychologically, early engagement fosters resilience by equipping children with adaptive strategies to cope with stress and failure (Jones et al., 2011). The transition to structured educational environments after preschool reinforces behavioural regularity (Dunn et al., 1994; Pedersen et al., 2007), positioning this phase as a pivotal opportunity to cultivate protective lifestyle habits that may offset early vulnerabilities. Consistent with this developmental framework, our study assessed the association between daily movement patterns at 7 years old and the subsequent incidence of self‐harm during adolescence (Boyden et al., 2019). Furthermore, compared with MVPA, increasing the time spent in LIPA may be more achievable and sustainable in real‐world settings. Prior observational studies have found that individuals with mental health difficulties are often unwilling or unable to engage in structured or high‐intensity physical activity (Stubbs et al., 2018). For physically inactive or insufficiently active children, encouraging movement of any intensity may be a more accessible and scalable approach. In this context, promoting LIPA represents a promising and pragmatic strategy. While MVPA remains a standard target for physical health, adherence can be challenging for adolescents with mental health difficulties or low fitness levels. Our findings suggest that LIPA offers a valuable ‘entry point’ for intervention. Replacing sedentary time with light activities (e.g. casual walking, light household tasks) is often more achievable and sustainable than immediate engagement in vigorous exercise for inactive youth. Consequently, teachers and primary care practitioners should consider advising families on integrating light physical activity into daily routines as a low‐risk, accessible, non‐pharmacological component of a holistic approach to mental wellness and self‐harm prevention.

In contrast to the straightforward linear protection observed with LIPA, the association between MVPA and self‐harm appears more complex. Our analysis revealed an inverted U dose–response relationship, specifically where intermediate levels of MVPA were associated with elevated risk compared with the protective effects seen at lower and higher levels. This distinct pattern may be explained by the varying contexts of physical activity. We speculate that intermediate durations of MVPA are more likely to reflect utilitarian or obligatory activities (e.g. active commuting to school, household chores). These activities possess strong ‘task attributes’; they are often solitary and physically demanding but lack enjoyment or social reward, potentially contributing to psychological stress or loneliness. Conversely, achieving high durations of MVPA typically necessitates engagement in organised competitive sports or team‐based activities. Unlike utilitarian tasks, these activities are often characterised by higher intensity and sustained bouts, which previous research suggests act as a proxy for structured after‐school sports rather than incidental movement (Miatke, Maher, Fraysse, Dumuid, & Olds, 2021). These structured activities are linked to strong ‘social attributes’, including peer support, social connectedness, and a sense of belonging, which likely buffer against mental distress.

### Strengths and limitations

Key strengths include the prospective, decade‐long design, which allowed for robust analysis of long‐term associations. The large, population‐based sample, derived from the Millennium Cohort Study, enhances the generalisability of the findings. The use of accelerometers to collect objective data on daily movement behaviours is another key strength, offering more reliable insights compared with self‐reported measures. Furthermore, the application of compositional logistic regression analysis, a sophisticated statistical approach, allows for a comprehensive evaluation of the interplay between various movement behaviours within a 24‐hr period. The study's results emphasise the significance of LIPA as a potential protective factor against self‐harm, a behaviour often overshadowed by the focus on MVPA in previous research. This novel contribution expands the understanding of how different movement behaviours collectively influence mental health outcomes. Notably, we observed dose–response relationships between movement behaviours and self‐harm risk. Specifically, a linear inverse association was observed for LIPA, while MVPA exhibited a non‐linear, inverted U‐shaped relationship with self‐harm risk. To the best of our knowledge, this is the first study to investigate the association between daily movement patterns and self‐harm using objective measures and CODA approach, providing an integrated perspective on how the interplay of these behaviours across a full 24‐hr cycle.

This study has limitations to consider. First, the measurement of sleep duration relied on subtracting parent‐reported non‐wear times (e.g. sleep, bathing) from accelerometer data, a method vulnerable to reporting inaccuracies that may systematically bias estimates compared with objective, continuous monitoring. Yet, given the relatively routine nature of children's behaviours, especially in the context of sleep, the self‐reported data are likely to be reasonably accurate. Second, although the Millennium Cohort Study employed rigorous sampling, its exclusion criteria (e.g. children with developmental disorders) and sociodemographic composition may limit generalisability to populations with distinct cultural sleep practices or health profiles. Third, a limitation of this study is the assumption that missing data were MAR, which may not fully hold, particularly for behavioural variables such as self‐harm. Furthermore, while we utilised a DAG to guide the selection of observed confounders based on prior knowledge (Figure S4), we acknowledge that residual confounding from unmeasured factors, such as genetic predispositions or specific adverse life events not captured in the dataset, remains a possibility. Nevertheless, according to the Bradford Hill criteria (Hill, 1965), the criteria of temporality and biological plausibility provide strong support for the association between LIPA and protection against self‐harm. Fourth, our study population, drawn from the UK Millennium Cohort, is predominantly of White ethnicity. We therefore incorporated a dichotomous categorisation (White vs. Other) for the analysis. This decision was necessitated by practical constraints, as sample sizes for distinct ethnic subgroups were insufficient to ensure statistical power in our multivariable models. We acknowledge that this approach limits our ability to explore heterogeneity within the ‘Other’ group and does not fully align with contemporary inclusivity guidelines (American Psychological Association [APA], 2019); however, it remained the most viable analytical strategy given these data limitations. As a result, our findings may not be generalisable to adolescents from other racial and ethnic backgrounds, who may experience different social and environmental stressors. Fifth, the strict inclusion criteria reduced the analytical sample to 2016 participants, which may have limited the representativeness and limited the generalisability of our findings. To address this concern, we compared the demographic characteristics of included and excluded participants; relevant details are provided in the Table S5. These limitations underscore the need for replication in diverse populations using more precise measurement approaches.

## Conclusion

Through the analysis of data derived from accelerometers, we identified a significant inverse association between LIPA and self‐harm behaviours, as well as a non‐linear relationship with MVPA, highlighting the nuanced roles of activity intensity and duration in shaping mental health outcomes. Our findings advocate for a holistic approach to child well‐being, emphasising the integration of physical activity into mental health initiatives to effectively mitigate self‐harm risks.

## Ethical considerations

The UK Millennium Cohort Study obtained ethical approval from the Law and Ethics Committee at the Institute of Education, University College London, and relevant local research ethics boards (approval dates: 2001–2018; protocol references available at https://cls.ucl.ac.uk/). Written informed consent was obtained from parents or legal guardians at each wave, and additional written assent was obtained from the adolescents at ages 14 and 17 years after the study procedures were fully explained. The present secondary analysis of de‐identified data was reviewed and approved by the Medical Ethics Committee of the Department of Psychological and Behavioural Sciences, Zhejiang University (Approval No. 2022060; approved June 2022).

**Key points**

**What's known?**

- Adolescent self‐harm is a major public health concern. While physical activity is thought to be protective, evidence is often inconsistent, reliant on self‐report, and primarily focused on MVPA.

**What's new?**

- Using objective accelerometer data from a large, decade‐long cohort, this study demonstrates a robust inverse association between LIPA and the incidence of adolescent self‐harm, a finding that is more pronounced than the non‐linear relationship observed for MVPA.

**What's relevant?**

- Promoting LIPA, a more accessible form of activity, represents a pragmatic and scalable public health strategy for early self‐harm prevention. Clinicians and educators can advise families on integrating more light activity into daily routines as a low‐risk component of mental health support.

## Supporting information


**Figure S1.** Consort diagram illustrating the flow of data in a longitudinal study based on the Millennium Cohort Study 2008–2017.
**Figure S2.** Sensitivity analyses of exploring alternative compositions and self‐harm risk across different behaviours in wave 6 (2015 year).
**Figure S3.** Sensitivity analyses exploring alternative compositions and self‐harm risk across different behaviours in wave 7 (2018 year).
**Figure S4.** Directed acyclic graph (DAG) guiding the selection of observed confounders.
**Table S1.** The compositional descriptive metrics for MVPA, LIPA, SB, and sleep duration including the compositional mean and log‐ratio.
**Table S2.** Compositional variation matrix of time spent in MVPA, LIPA, SB, and sleep duration.
**Table S3.** Sensitivity analyses of single predictor and compositional (CODA) models of how time allocated to a single behaviour affects self‐harm in MCS6, MCS7 waves for each behaviour.
**Table S4.** Sensitivity analyses of compositional model coefficient estimates and 95% confidence interval in MCS6, MCS7 waves.
**Table S5.** Comparison of characteristics between the analytic sample and the excluded sample (*n* = 8,939).
**Appendix S1.** Calculation of compositional mean.
**Appendix S2.** Explanation of variation matrix.
**Appendix S3.** Calculation of log‐ratio difference.
**Appendix S4.** Explanation of CODA model and compositional logistic regression model.
**Appendix S5.** Explanation for the ternary plots.
**Appendix S6.** The selection of confounders.

## Data Availability

The data that support the findings of this study are available from Centre for Longitudinal Studies (CLS). Restrictions apply to the availability of these data, which were used under licence for this study. Data are available from https://ukdataservice.ac.uk/ with the permission of Centre for Longitudinal Studies (CLS).

## References

[jcpp70199-bib-0001] Aitchison, J. (1982). The statistical analysis of compositional data. Journal of the Royal Statistical Society. Series B, Methodological, 44, 139–177.

[jcpp70199-bib-0002] American Psychological Association . (2019). APA guidelines on race and ethnicity in psychology. Available from: https://www.apa.org/about/policy/guidelines‐race‐ethnicity.pdf. Accessed 17 September 2024.

[jcpp70199-bib-0003] Biddle, S.J.H. , Ciaccioni, S. , Thomas, G. , & Vergeer, I. (2019). Physical activity and mental health in children and adolescents: An updated review of reviews and an analysis of causality. Psychology of Sport and Exercise, 42, 146–155.

[jcpp70199-bib-0004] Blanchard, A. , Chihuri, S. , DiGuiseppi, C.G. , & Li, G. (2021). Risk of self‐harm in children and adults with autism spectrum disorder: A systematic review and meta‐analysis. JAMA Network Open, 4, e2130272. 10.1001/jamanetworkopen.2021.30272 PMC8527356 34665237

[jcpp70199-bib-0005] Boyden, J. , Dawes, A. , Dornan, P. , & Tredoux, C. (Eds.). (2019). Middle childhood: A key time for healthy development and learning. Bristol: Policy Press.

[jcpp70199-bib-0006] Butte, N.F. , Watson, K.B. , Ridley, K. , Zakeri, I.F. , McMurray, R.G. , Pfeiffer, K.A. , … & Fulton, J.A.N.E.T.E. (2018). A youth compendium of physical activities: Activity codes and metabolic intensities. Medicine and Science in Sports and Exercise, 50, 246–256. 10.1249/MSS.0000000000001430 PMC5768467 28938248

[jcpp70199-bib-0007] Chastin, S.F.M. , Palarea‐Albaladejo, J. , Dontje, M.L. , & Skelton, D.A. (2015). Combined effects of time spent in physical activity, sedentary behaviors and sleep on obesity and cardio‐metabolic health markers: A novel compositional data analysis approach. PLoS One, 10, e0139984. 10.1371/journal.pone.0139984 PMC4604082 26461112

[jcpp70199-bib-0008] Chen, Z. , Xin, J. , Jia, J. , Tu, J. , & Li, X. (2025). The association between weekend warrior physical activity pattern and anxiety: Evidence from a US population‐based study. BMC Psychiatry, 25, 168. 10.1186/s12888-025-06612-x PMC11863453 40001092

[jcpp70199-bib-0009] Connelly, R. , & Platt, L. (2014). Cohort profile: UK millennium cohort study (MCS). International Journal of Epidemiology, 43, 1719–1725. 10.1093/ije/dyu001 24550246

[jcpp70199-bib-0010] Desquilbet, L. , & Mariotti, F. (2010). Dose‐response analyses using restricted cubic spline functions in public health research. Statistics in Medicine, 29, 1037–1057. 10.1002/sim.3841 20087875

[jcpp70199-bib-0011] Dunn, J. , Slomkowski, C. , & Beardsall, L. (1994). Sibling relationships from the preschool period through middle childhood and early adolescence. Developmental Psychology, 30, 315–324. 10.1111/j.1469-7610.1994.tb01736.x 8195308

[jcpp70199-bib-0012] Evenson, K.R. , Catellier, D.J. , Gill, K. , Ondrak, K.S. , & McMurray, R.G. (2008). Calibration of two objective measures of physical activity for children. Journal of Sports Sciences, 26, 1557–1565. 10.1080/02640410802334196 18949660

[jcpp70199-bib-0013] Felez‐Nobrega, M. , Bort‐Roig, J. , Ma, R. , Romano, E. , Faires, M. , Stubbs, B. , … & Koyanagi, A. (2021). Light‐intensity physical activity and mental ill health: A systematic review of observational studies in the general population. International Journal of Behavioral Nutrition and Physical Activity, 18, 149. 10.1186/s12966-021-01196-7 PMC8444599 34526048

[jcpp70199-bib-0014] Füzéki, E. , Engeroff, T. , & Banzer, W. (2017). Health benefits of light‐intensity physical activity: A systematic review of accelerometer data of the National Health and Nutrition Examination Survey (NHANES). Sports Medicine, 47, 1769–1793. 10.1007/s40279-017-0724-0 28393328

[jcpp70199-bib-0015] Gao, Y. , Lu, C. , Zhang, X. , Han, B. , & Hu, H. (2024). Physical activity and non‐suicidal self‐injurious behavior in Chinese adolescents: The chain mediating role of psychological capital and relative deprivation. Frontiers in Psychiatry, 15, 1509967. 10.3389/fpsyt.2024.1509967 PMC11638173 39676912

[jcpp70199-bib-0016] Geraci, M. , Rich, C. , Sera, F. , Cortina‐Borja, M. , & Griffiths, L.J. (2012). Technical report on accelerometry data processing in the Millennium Cohort Study. London: University College London.

[jcpp70199-bib-0017] Greenacre, M. (2021). Compositional data analysis. Annual Review of Statistics and Its Application, 8, 271–299.

[jcpp70199-bib-0018] Hawton, K. , Saunders, K.E. , & O'Connor, R.C. (2012). Self‐harm and suicide in adolescents. The Lancet, 379, 2373–2382. 10.1016/S0140-6736(12)60322-5 22726518

[jcpp70199-bib-0019] Hayes, G. , Dowd, K.P. , MacDonncha, C. , & Donnelly, A.E. (2019). Tracking of physical activity and sedentary behavior from adolescence to young adulthood: A systematic literature review. Journal of Adolescent Health, 65, 446–454. 10.1016/j.jadohealth.2019.03.013 31248803

[jcpp70199-bib-0051] Hill, A.B. (1965). The environment and disease: Association or causation?. Proceedings of the Royal Society of Medicine, 58, 295–300. 10.1177/003591576505800503 PMC1898525 14283879

[jcpp70199-bib-0020] Hron, K. , Filzmoser, P. , & Thompson, K. (2012). Linear regression with compositional explanatory variables. Journal of Applied Statistics, 39, 1115–1128.

[jcpp70199-bib-0021] Jarvi, S.M. , Hearon, B.A. , Batejan, K.L. , Gironde, S. , & Björgvinsson, T. (2017). Relations between past‐week physical activity and recent nonsuicidal self‐injury in treatment‐seeking psychiatric adults. Journal of Clinical Psychology, 73, 479–488. 10.1002/jclp.22342 27391124

[jcpp70199-bib-0022] Jones, A.C. , Schinka, K.C. , van Dulmen, M.H. , Bossarte, R.M. , & Swahn, M.H. (2011). Changes in loneliness during middle childhood predict risk for adolescent suicidality indirectly through mental health problems. Journal of Clinical Child & Adolescent Psychology, 40, 818–824. 10.1080/15374416.2011.614585 22023273

[jcpp70199-bib-0023] Jung, J. , Lee, S. , Lee, J.H. , & Lee, D. (2025). Associations between physical activities and self‐harm behaviour in depression across the genotype: Findings from the UK Biobank. BJPsych Open, 11, e27. 10.1192/bjo.2024.845 PMC11822987 39885769

[jcpp70199-bib-0024] Knafo‐Noam, A. , Daniel, E. , & Benish‐Weisman, M. (2024). The development of values in middle childhood: Five maturation criteria. Current Directions in Psychological Science, 33, 18–26.

[jcpp70199-bib-0025] Laflamme, L. , Vaez, M. , Lundin, K. , & Sengoelge, M. (2022). Prevention of suicidal behavior in older people: A systematic review of reviews. PLoS One, 17, e0262889. 10.1371/journal.pone.0262889 PMC8789110 35077476

[jcpp70199-bib-0026] Lai, S. , Su, C. , Song, S. , Yan, M. , Tang, C. , Zhang, Q. , … & Liu, Q. (2021). Depression and deliberate self‐harm among rural adolescents of Sichuan Province in Western China: A 2‐year longitudinal study. Frontiers in Psychiatry, 12, 605785. 10.3389/fpsyt.2021.605785 PMC8473622 34589002

[jcpp70199-bib-0027] Latina, D. , Jaf, D. , Alberti, R. , & Tilton‐Weaver, L. (2022). Can participation in organised sports help adolescents refrain from self‐harm? An analysis of underlying mechanisms. Psychology of Sport and Exercise, 59, 102133.

[jcpp70199-bib-0028] Liu, M. , Zhang, J. , Kamper‐DeMarco, K.E. , Hu, E. , & Yao, S. (2020). Associations of moderate‐to‐vigorous physical activity with psychological problems and suicidality in Chinese high school students: A cross‐sectional study. PeerJ, 8, e8775. 10.7717/peerj.8775 PMC7102502 32257640

[jcpp70199-bib-0029] Love, R. , Adams, J. , Atkin, A. , & van Sluijs, E. (2019). Socioeconomic and ethnic differences in children's vigorous intensity physical activity: A cross‐sectional analysis of the UK Millennium Cohort Study. BMJ Open, 9, e027627. 10.1136/bmjopen-2018-027627 PMC6549689 31133593

[jcpp70199-bib-0030] Lucena, N.L. , Rossi, T.A. , Azevedo, L.M.G. , & Pereira, M. (2022). Self‐injury prevalence in adolescents: A global systematic review and meta‐analysis. Children and Youth Services Review, 142, 106634.

[jcpp70199-bib-0031] Maciejewski, D.F. , Creemers, H.E. , Lynskey, M.T. , Madden, P.A. , Heath, A.C. , Statham, D.J. , … & Verweij, K.J.H. (2014). Overlapping genetic and environmental influences on nonsuicidal self‐injury and suicidal ideation: Different outcomes, same etiology? JAMA Psychiatry, 71, 699–705. 10.1001/jamapsychiatry.2014.89 PMC4241464 24760386

[jcpp70199-bib-0032] Masquelier, B. , Hug, L. , Sharrow, D. , You, D. , Mathers, C. , Gerland, P. , … & UN Inter‐agency Group for Child Mortality Estimation . (2021). Global, regional, and national mortality trends in youth aged 15‐24 years between 1990 and 2019: A systematic analysis. The Lancet Global Health, 9, e409–e417. 10.1016/S2214-109X(21)00023-1 PMC7966666 33662320

[jcpp70199-bib-0033] McGregor, D.E. , Palarea‐Albaladejo, J. , Dall, P.M. , Hron, K. , & Chastin, S.F.M. (2020). Cox regression survival analysis with compositional covariates: Application to modelling mortality risk from 24‐h physical activity patterns. Statistical Methods in Medical Research, 29, 1447–1465. 10.1177/0962280219864125 31342855

[jcpp70199-bib-0034] Medeiros, M.L. , da Costa, A.D. , Vieira, C.J. , Silva, P.H. , Santos, D.A. , & Vitalle, M.S.S. (2025). Sedentary behavior and self‐harm in adolescents with asthma, rhinitis and eczema. Jornal de Pediatria, 101, 202–208. 10.1016/j.jped.2024.08.003 PMC11889675 39245234

[jcpp70199-bib-0035] Miatke, A. , Maher, C. , Fraysse, F. , Dumuid, D. , & Olds, T. (2021). Are all MVPA minutes equal? Associations between MVPA characteristics, independent of duration, and childhood adiposity. BMC Public Health, 21, 1321. 10.1186/s12889-021-11420-5 PMC8259325 34225692

[jcpp70199-bib-0036] O'Reilly, L. , Elam, K.K. , Quinn, P.D. , Adams, S. , Chirica, M.G. , Klonsky, E.D. , … & D'Onofrio, B. (2022). Examining protective factors for substance use problems and self‐harm behavior during adolescence: A longitudinal co‐twin control study. Development and Psychopathology, 34, 1781–1802. 10.1017/S0954579422000724 PMC9929025 35968852

[jcpp70199-bib-0037] Ougrin, D. , & Boege, I. (2013). Brief report: The Self Harm Questionnaire: A new tool designed to improve identification of self harm in adolescents. Journal of Adolescence, 36, 221–225. 10.1016/j.adolescence.2012.09.006 23046733

[jcpp70199-bib-0038] Pagani, L.S. , Fitzpatrick, C. , Barnett, T.A. , & Dubow, E. (2010). Prospective associations between early childhood television exposure and academic, psychosocial, and physical well‐being by middle childhood. Archives of Pediatrics & Adolescent Medicine, 164, 425–431. 10.1001/archpediatrics.2010.50 20439793

[jcpp70199-bib-0039] Pedersen, S. , Vitaro, F. , Barker, E.D. , & Borge, A.I.H. (2007). The timing of middle‐childhood peer rejection and friendship: Linking early behavior to early‐adolescent adjustment. Child Development, 78, 1037–1051. 10.1111/j.1467-8624.2007.01051.x 17650124

[jcpp70199-bib-0040] Pulsford, R.M. , Cortina‐Borja, M. , Rich, C. , Kinnafick, F.E. , Dezateux, C. , & Griffiths, L.J. (2011). Actigraph accelerometer‐defined boundaries for sedentary behaviour and physical activity intensities in 7 year old children. PLoS One, 6, e21822. 10.1371/journal.pone.0021822 PMC3154898 21853021

[jcpp70199-bib-0041] Shan, D. , Wang, Y. , Tousey‐Pfarrer, M. , Guo, C. , Wan, M. , Wang, P. , … & Zhang, J. (2024). Association between patterns of biological rhythm and self‐harm: Evidence from the Baoxing youth mental health (BYMH) cohort. Child and Adolescent Psychiatry and Mental Health, 18, 3. 10.1186/s13034-023-00685-w PMC10765742 38172979

[jcpp70199-bib-0042] Sokolowski, M. , Wasserman, J. , & Wasserman, D. (2020). Genetic aetiology of self‐harm ideation and behaviour. Nature Communications, 11, 4748.

[jcpp70199-bib-0043] Stubbs, B. , Vancampfort, D. , Hallgren, M. , Firth, J. , Veronese, N. , Solmi, M. , … & Kahl, K.G. (2018). EPA guidance on physical activity as a treatment for severe mental illness: A meta‐review of the evidence and position statement from the European Psychiatric Association (EPA), supported by the International Organization of Physical Therapists in Mental Health (IOPTMH). European Psychiatry, 54, 124–144. 10.1016/j.eurpsy.2018.07.004 30257806

[jcpp70199-bib-0044] Telama, R. , Yang, X. , Leskinen, E. , Kankaanpää, A. , Hirvensalo, M. , Tammelin, T. , … & Raitakari, O.T. (2014). Tracking of physical activity from early childhood through youth into adulthood. Medicine & Science in Sports & Exercise, 46, 955–962. 10.1249/MSS.0000000000000181 24121247

[jcpp70199-bib-0045] Tennant, P.W. , Murray, E.J. , Arnold, K.F. , Berrie, L. , Fox, M.P. , Gadd, S.C. , … & Ellison, G.T. (2021). Use of directed acyclic graphs (DAGs) to identify confounders in applied health research: Review and recommendations. International Journal of Epidemiology, 50, 620–632. 10.1093/ije/dyaa213 PMC8128477 33330936

[jcpp70199-bib-0046] van Buuren, S. , & Groothuis‐Oudshoorn, K. (2011). Mice: Multivariate imputation by chained equations in R. Journal of Statistical Software, 45, 1–67.

[jcpp70199-bib-0047] Yang, L. , Jia, F.F. , Lu, X.Y. , & Jia, C.X. (2023). Internalizing and externalizing problems mediate the relationship between maltreatment and self‐harm among UK adolescents. Journal of Affective Disorders, 333, 240–248. 10.1016/j.jad.2023.04.046 37084976

[jcpp70199-bib-0048] Yu, H. , Mu, Q. , & Li, K. (2023). Effects of physical exercise on non‐suicidal self‐injury in adolescents: The chain mediating role of perceived social support and self‐concept. Frontiers in Psychiatry, 14, 1201863. 10.3389/fpsyt.2023.1201863 PMC10591106 37876620

[jcpp70199-bib-0049] Zhang, Y. , Wang, Z. , Zhang, R. , Wang, Y. , Yang, J. , Wang, F. , & Liu, Z. (2025). Non‐linear dose‐response association between physical activity and mental health in adolescents: A prospective cohort study based on SEARCH. International Journal of Behavioral Nutrition and Physical Activity, 22, 146. 10.1186/s12966-025-01848-y PMC12632063 41267100

[jcpp70199-bib-0050] Zhao, H. , He, L. , Liu, C. , Shan, X. , Gui, C. , Zhang, L. , … & Luo, B. (2024). Self‐harm and interpersonal violence due to high temperature from the Global Burden of Disease Study 2019: A 30‐year assessment. Environmental Research, 243, 117826. 10.1016/j.envres.2023.117826 38081341

