# Supplementary material for: The association between 24‐hr movement patterns and self‐harm incidence in adolescence
Source: J Child Psychol Psychiatry. 2026 Jul 9;67(12):2019–30. doi: 10.1111/jcpp.70199 (PMC13745792; doi:10.1111/jcpp.70199)
Supplement: Supplementary file 1 [file JCPP-67-2019-s001.docx]

**The Association Between 24-Hour Movement Patterns and Self-Harm Incidence in Adolescence**

**Supporting Information**

**Table S1. The compositional descriptive metrics for MVPA, LIPA, SB and sleep duration including the compositional mean and log-ratio**

|  | **Overall (N=2016)** | **Non-self-harm (N=1468)** | **Self-harm(N=548)** |
| --- | --- | --- | --- |
| **Compositional mean (hours/day) ^a^** | | | |
| MVPA | 1.14 | 1.15 | 1.10 |
| LIPA | 4.75 | 4.79 | 4.66 |
| SB | 6.69 | 6.68 | 6.74 |
| Sleep | 11.43 | 11.38 | 11.51 |
| **Log-ratio (group compositional mean/total sample compositional mean) as % ^b^** | | | |
| MVPA | 0 | 0.87 | -3.57 |
| LIPA | 0 | 0.84 | -1.91 |
| SB | 0 | -0.15 | 0.60 |
| Sleep | 0 | -0.35 | 0.79 |

^a^ The compositional mean was obtained by computed the proportion of daily time spent in each behavior and transforming it back to the 24-hour scale.

^b^ The relative time spent on each behavior is expressed as the logarithmic ratio of the compositional mean for each group to the full sample compositional mean multiplied by 100 (%).

**Table S2. Compositional variation matrix of time spent in MVPA, LIPA, SB and sleep duration**

|  | **MVPA** | **LIPA** | **SB** | **SLEEP** |
| --- | --- | --- | --- | --- |
| **MVPA** | 0.00 | 0.13 | 0.24 | 0.17 |
| **LIPA** | 0.13 | 0.00 | 0.07 | 0.05 |
| **SB** | 0.24 | 0.07 | 0.00 | 0.07 |
| **Sleep** | 0.17 | 0.05 | 0.07 | 0.00 |

**Table S3. Sensitivity analyses of Single Predictor and compositional (CODA) models of how time allocated to a single behavior affects self-harm in MCS6, MCS7 waves for each behavior**

| **Wave** | **Behaviors** | **SINGLE ^a^** | | **CODA ^b^** | |
| --- | --- | --- | --- | --- | --- |
|  |  | **OR (95%CI)** | ***P-*values** | **OR (95%CI)** | ***P*-values** |
| **MCS6** | MVPA | 0.97 (0.70-1.33) | 0.84 | 1.06 (0.71-1.58) | 0.79 |
|  | LIPA | 0.81 (0.66-0.99) | **0.04** | 0.33 (0.13-0.84) | **0.02** |
|  | Sleep | 1.12 (0.98-1.29) | 0.12 | 1.60 (0.73-3.58) | 0.25 |
|  | SB | 0.99 (0.88-1.11) | 0.91 | 1.05 (0.62-1.78) | 0.86 |
| **MCS7** | MVPA | 0.87 (0.66-1.13) | 0.30 | 0.96 (0.69-1.35) | 0.81 |
|  | LIPA | 0.77 (0.65-0.91) | **0.002** | 0.29 (0.13-0.63) | **0.002** |
|  | Sleep | 1.03 (0.93-1.15) | 0.55 | 1.27 (0.67-2.44) | 0.47 |
|  | SB | 1.07 (0.98-1.17) | 0.12 | 1.42 (0.92-2.21) | 0.12 |

MVPA, Moderate-vigorous physical activity; LIPA, Light-intensity physical activity; SB, sedentary behaviour.

Single Predictor and CODA models adjusted for age, sex, ethnic, body mass index (BMI) z-score, residence, family income, children’s mental disorder, children’s interpersonal difficulties, parents’ mental disorder and parents’ marriage status.

^a^ SINGLE model: The exposure variable was used to assess the univariate relationship between each behavior and the outcome by evaluating the time allocated to a single behavior.

^b^ CODA model: The regression coefficient reflected the variation in the log-ratio of a specific behavior relative to the others.

**Table S4. Sensitivity analyses of compositional model coefficient estimates and 95% confidence interval in MCS6, MCS7 waves**

| **Wave** | **ILR-coordinates ^a^** | **OR (95%CI) ^b^** | ***P*-values ^c^** |
| --- | --- | --- | --- |
| **MCS6** | *Z*1 | 0.30 (0.12-0.77) | **0.01** |
|  | *Z*2 | 0.85 (0.56-1.30) | 0.46 |
|  | *Z*3 | 1.56 (0.67-3.89) | 0.33 |
|  | Overall | - | 0.09 |
|  | Hoslem_test |  | 0.12 |
| **MCS7** | *Z*1 | 0.27 (0.12-0.61) | **0.001** |
|  | *Z*2 | 0.84 (0.59-1.20) | 0.33 |
|  | *Z*3 | 0.90 (0.47-1.78) | 0.75 |
|  | Overall | - | **0.01** |
|  | Hoslem_test |  | 0.45 |

^a^ *Z* was defined as: $Z=(\sqrt{\frac{3}{4}}ln\frac{LIPA}{\left( Sleep\cdot SB\cdot MVPA \right)^{1/3}},\sqrt{\frac{2}{3}}ln\frac{MVPA}{\left( Sleep\cdot SB \right)^{1/2}},\sqrt{\frac{1}{2}}ln\frac{Sleep}{SB})$

^b^ OR indicated change in odds ratio per unit increase in the associated ILR-coordinates. Model adjusted for age, sex, ethnic, body mass index (BMI) z-score, residence, family income, children’s mental disorder, children’s interpersonal difficulties, parents’ mental disorder and parents’ marriage status.

^c^ *P*-values for individual ILR-coordinates were based on Wald tests and overall *P*-values were based on likelihood ratio tests (LRT).

**Table S5. Comparison of characteristics between the analytic sample and the excluded sample (n=8939)**

|  | **Overall (N=8939)** | **Excluded Sample (N=6923)** | **Analytic Sample (N=2016)** | ***P*-values ^a^** |
| --- | --- | --- | --- | --- |
| **Age (mean (SD))** | 7.23 (0.25) | 7.23 (0.25) | 7.23 (0.25) | 0.612 |
| **Sex, n (%)** |  |  |  | **<0.001** |
| Male | 4257 (47.6) | 3222 (46.5) | 1035 (51.3) |  |
| Female | 4317 (48.3) | 3336 (48.2) | 981 (48.7) |  |
| Missing | 365 (4.1) | 365 (5.3) | 0 (0.0) |  |
| **Ethic, n (%)** |  |  |  | **0.003** |
| White | 1289 (14.4) | 1017 (14.7) | 272 (13.5) |  |
| Mixed ^b^ | 7618 (85.2) | 5874 (84.8) | 1744 (86.5) |  |
| Missing | 32 (0.4) | 32 (0.5) | 0 (0.0) |  |
| **Residence, n (%)** |  |  |  | 0.246 |
| Urban > 10K | 5628 (63.0) | 4382 (63.3) | 1246 (61.8) |  |
| Town and Fringe | 682 (7.6) | 519 (7.5) | 163 (8.1) |  |
| Village, Hamlet & Isolated Dwellings | 677 (7.6) | 506 (7.3) | 171 (8.5) |  |
| Missing | 1952 (21.8) | 1516 (21.9) | 436 (21.6) |  |
| **BMI (kg/m^2^) (mean (SD))** | 16.53 (2.24) | 16.59 (2.31) | 16.31 (1.97) | **<0.001** |
| **BMI z-score (mean (SD))** | 0.69 (1.46) | 0.73 (1.50) | 0.56 (1.29) | **<0.001** |
| **Family income (mean (SD))** | 407.13 (229.29) | 395.53 (227.09) | 446.83 (232.36) | **<0.001** |
| **Parents’ marriage status, n (%)** |  |  |  | **<0.001** |
| Legally Separated ^c^ | 2464 (27.6) | 2022 (29.2) | 442 (21.9) |  |
| Married | 6239 (69.8) | 4703 (67.9) | 1536 (76.2) |  |
| Missing | 236 (2.6) | 198 (2.9) | 38 (1.9) |  |
| **Parents’ mental disorder, n (%)** |  |  |  | 0.068 |
| History diagnosed with depression/serious anxiety | 7416 (83.0) | 5709 (82.5) | 1707 (84.7) |  |
| No history of depression or serious anxiety diagnosis | 1207 (13.5) | 962 (13.9) | 245 (12.2) |  |
| Missing | 316 (3.5) | 252 (3.6) | 64 (3.2) |  |

BMI, body mass index; SD, standard deviation;

^a^ Continuous and categorical variables were compared using analyses of variance and χ^2^ tests, respectively

^b^ Indicated Mixed/Indian/ Pakistani and Bangladeshi/ Pakistani and Bangladeshi/ Another ethnic group

^c^ Indicated Legally Separated/Single/Divorced/Widowed

**Figure S1. Consort diagram illustrating the flow of data in a longitudinal study based on the Millennium Cohort Study 2008-2017.**

Note: "Lost to follow-up" refers to participants who did not provide outcome data (self-harm) in either the 2015 (age 14) or 2018 (age 17) waves. To be included in the analytic sample, participants were required to have valid accelerometer data at baseline and at least one valid outcome measurement in either 2015 or 2018. Multiple imputation was performed to address missing data in baseline covariates for those participants included in the analytic sample.

**Figure S2. Sensitivity analyses of exploring alternative compositions and self-harm risk across different behaviors in wave 6 (2015 year)**

These plots explore the impact of reallocating time to or from one behaviour relative to the others on self-harm risk. (a) Moderate-vigorous physical activity; (b) Light-intensity physical activity; (c) Sedentary behavior; and (d) Sleep duration. The intersection point of the lines represents the sample compositional mean, which serves as the reference: 68 min (1.14 h) of MVPA, 285 min (4.75 h) of LIPA, 402 min (6.69 h) of SB, and 685 min (11.42 h) of sleep duration.

**Figure S3. Sensitivity analyses exploring alternative compositions and self-harm risk across different behaviors in wave 7 (2018 year).**

These plots explore the impact of reallocating time to or from one behaviour relative to the others on self-harm risk. (a) Moderate-vigorous physical activity; (b) Light-intensity physical activity; (c) Sedentary behavior; and (d) Sleep duration. The intersection point of the lines represents the sample compositional mean, which serves as the reference: 68 min (1.14 h) of MVPA, 285 min (4.75 h) of LIPA, 402 min (6.69 h) of SB, and 685 min (11.42 h) of sleep duration.

**Supporting Appendices**

**Appendix S1. Calculation of Compositional Mean**

In the descriptive analysis, the compositional mean was used to illustrate the differences in MVPA, LIPA, SB, and sleep between the groups with and without self-harm behaviors. The calculation method for the compositional mean is as follows:

**Compositional Mean**

This explanation is describing a statistical method called compositional mean, which is used to analyze data that represents parts of a whole (e.g., the relative distribution of different behaviors within a fixed total, such as 24 hours). Here's a breakdown of what the explanation is saying:

(1) Compositional Mean and Relative Distribution

Compositional Mean is a method used to adjust and analyze the relative distribution of different behaviors (or components) in a fixed total. In this context, it refers to the relative time spent in different behaviors within a 24-hour period (e.g., sleep, physical activity, sedentary behavior, etc.).

The key idea here is that the sum of these behaviors must always equal a constant (in this case, 24 hours). Therefore, the method adjusts the data so that it expresses each behavior as a proportion of the total time, allowing for comparison between the different behaviors.

(2) Formula Explanation

The formula provided is:

$$\mathrm{Cen}=\frac{g_{i}}{C(g_{1}, \ldots,g_{i})}$$

where: Cen represents the compositional mean for the 𝑖-th behaviour.

g𝑖 is the geometric mean of the time spent on the 𝑖-th behavior (for example, the average time spent sleeping, sitting, or engaging in physical activity).

C is the closure operator. This operator "closes" the data by transforming it into a form where the sum of all components equals a constant (here, 1 or 100%, depending on how the data is scaled).

(3) Closure Operator (C)

The closure operation ensures that the data adds up to a constant total. In practice, this means dividing each component by the sum of all components, so that each behavior is represented as a proportion of the total. For instance:

If you have four behaviors and the total time is 24 hours, each behavior is represented as a proportion of the total 24 hours.

The closure operator makes sure that the sum of the proportions for all behaviors equals 1 (or 100%).

(4) Geometric Mean of Behaviors

The geometric mean 𝑔𝑖 is calculated for each behavior (e.g., time spent sleeping, sitting, or physical activities). The geometric mean is often used in this context because it is less sensitive to extreme values and provides a more stable measure when dealing with proportional data.

(5) Putting It All Together

The process involves:

a. Computing the geometric mean for the time spent on each behavior.

b. Applying the closure operator, so that all the behaviors sum to 1 or 100%, thus turning the raw times into proportions.

c. The resulting data represents the relative distribution of each behavior in relation to the others, allowing for a more accurate comparison of how time is allocated across behaviors within the fixed total of 24 hours.

In Summary: This approach is used to understand how different activities (such as physical activity, sedentary behavior, and sleep) are proportionally distributed within a fixed time frame (like 24 hours). By computing the compositional mean, the method adjusts the data to reflect the relative distribution of these behaviors, considering their interdependence and ensuring they sum up to a constant total. The geometric mean is used to calculate each behavior's central tendency, and the closure operator ensures the data is properly scaled.

Reference: Pedišić Ž. Measurement issues and poor adjustments for physical activity and sleep undermine sedentary behaviour research—the focus should shift to the balance between sleep, sedentary behaviour, standing and activity[J]. Kinesiology, 2014.

Chastin SFM, Palarea-Albaladejo J. Concise Guide to Compositional Data Analysis for Physical Activity, Sedentary Behavior and Sleep Research: Supplementary Material S2, in Chastin SFM, Palarea-Albaladejo J, Dontje ML, Skelton DA. “Combined effects of time spent in physical activity, sedentary behavior and sleep on adiposity and cardiometabolic health markers: a novel compositional data analysis approach.

**Appendix S2. Explanation of Variation Matrix**

A variation matrix was utilized to illustrate the relationships/co-dependence among these daily movements. The variability of the data was summarized using a variation matrix, which contains the pairwise log-ratio variances for each pair of movements. This matrix allows us to assess the degree of association between different movements. A value close to zero in the matrix indicates that the times spent on the two movements involved in the ratio (arranged by rows and columns) are highly co-dependence to each other.

**Appendix S3. Calculation of Log-Ratio Difference:**

The log-ratio difference is a method used to quantify the relative differences between the proportions of different components (e.g., behaviors such as MVPA, LIPA, SB, and sleep) between two groups of interest. This approach allows us to capture the interdependencies between behaviors and emphasize the relative differences between groups.

The formula used for calculating the log-ratio difference is:

$$log-ratio difference =100\ln\left( \frac{{Cen}_{i}}{Cen} \right)(\%)$$

Where:

- Ceni represents the compositional mean for a specific group iii, such as the group with self-harm or the group without self-harm.
- Cen represents the overall compositional mean calculated for the entire population (or for a reference group, depending on the study design).

Steps in Log-Ratio Difference Calculation:

(1) Compute Compositional Mean for Each Group: First, the compositional mean (Cen) is calculated separately for each group of interest. For example, one calculation for individuals with self-harm and another for individuals without self-harm. The compositional mean reflects the relative distribution of time across different behaviors (MVPA, LIPA, SB, Sleep) within each group.
(2) Calculate the Log-Ratio for Each Behavior: For each behavior (e.g., MVPA, LIPA, SB, and Sleep), the log-ratio difference is calculated by comparing the compositional mean of each group (Ceni) with the overall compositional mean (Cen). The natural logarithm (ln) is used to ensure that the ratio is symmetric and continuous, allowing us to capture the relative magnitude of differences in both directions (whether one behavior is more or less prominent in a group compared to the overall mean).
(3) Interpretation of the Log-Ratio: The log-ratio gives a measure of how much more or less time is allocated to a given behavior in one group compared to the overall population (or reference group). A positive log-ratio indicates that the group spends more time on that behavior relative to the overall population, while a negative log-ratio indicates less time spent on that behavior. The result is then multiplied by 100 to express the log-ratio difference as a percentage, making it easier to interpret and visualize.

**Appendix S4. Explanation of CODA model and Compositional logistic regression model**

To account for the interactions between the four movement behaviors, we performed an isometric log-ratio (ILR) transformation on the data before incorporating it into the CODA model. After the transformation, the sum of the transformed values for the four movements (MVPA, LIPA, sedentary behavior, and sleep) equals one. In the model, we included the transformed value of a specific movement, alongside the sum of the remaining three movements. This approach allowed us to examine the association between each specific movement and self-harm behaviors, while controlling for the influence of the other three behaviors.

As shown in Table 2, the ILR for LIPA vs. the remaining behaviors demonstrated a statistically significant association (p < 0.001), indicating that allocating more time to LIPA is associated with a reduced risk of self-harm. Following this, we constructed a ternary coordinate system (Z = Z1, Z2, Z3), where Z1 represents the LIPA-vs-remaining ILR, allowing us to explore its relationship with self-harm. Subsequently, we examined the impact of the remaining three behaviors on self-harm. Specifically, Z2 represents the transformed value for MVPA, while controlling for LIPA, and the sum of the remaining two behaviors' transformed values. Similarly, Z3 explores the relationship between self-harm and the transformed values of sleep and SB, after controlling for MVPA.

$$Z=(\sqrt{\frac{3}{4}}ln\frac{LIPA}{\left( MVPA\cdot S\cdot SB \right)^{1/3}},\sqrt{\frac{2}{3}}ln\frac{MVPA}{\left( S\cdot SB \right)^{1/2}},\sqrt{\frac{1}{2}}ln\frac{S}{SB})$$

*Z1*: The ratio of LIPA time in relation to time in MVPA, SB and SLEEP.

*Z2:* The ratio of MVPA time in relation to time in SB and SLEEP.

*Z3:* The ratio of SB time in relation to time in SLEEP.

The ILR-coordinates of the triad were used to construct a model of the effect on the outcome after substituting one behavior for another while holding the other two behaviors constant through isotemporal analysis. Odds ratios and 95% CIs were calculated using the Monte Carlo method combined with the coefficient distribution from the generalized linear model (GLM). The rmvnorm function generated a set of 100,000 possible coefficients from a multivariate normal distribution to model uncertainty. Then, multiple possible ORs were obtained using GLM. The mean and quantiles of the OR samples were calculated, with the 2.5% and 97.5% quantiles forming the 95% CI.

Reference:

Hron K, Filzmoser P, Thompson K. Linear regression with compositional explanatory variables. J Appl Stat. Taylor & Francis; 2012; 39: 1115–1128. doi: 10.1080/02664763.2011.644268

Chastin SFM, Palarea-Albaladejo J, Dontje ML, Skelton DA. Combined Effects of Time Spent in Physical Activity, Sedentary Behaviors and Sleep on Obesity and Cardio-Metabolic Health Markers: A Novel Compositional Data Analysis Approach. PLOS ONE. 2015;10(10):e0139984.

McGregor D, Palarea-Albaladejo J, Dall P, et al. Cox regression survival analysis with compositional covariates: Application to modelling mortality risk from 24-h physical activity patterns[J]. Statistical Methods in Medical Research, 2020, 29(5): 1447-1465.

**Appendix S5. Explanation for the ternary plots:**

Ternary plots were used to predict the expected odds ratios (ORs) based on the compositional means of the four behaviors (MVPA, LIPA, SB, and sleep), with the observed values in the dataset as the reference composition: (MVPA, 1.14; LIPA, 4.75; SB, 6.69; sleep, 11.42 hours). Since ternary plots have only three dimensions，in each plot, one behavior is fixed, and the expected OR is shown for a range of combinations of the remaining three behaviors. Given the four behaviors under study (MVPA, LIPA, SB, and sleep), four separate ternary plots were generated, each excluding one behavior to visualize the interactions among the other three. This approach allows for the exploration of all possible combinations and their joint effects. In the heatmaps, we observe that when LIPA is included as a variable, as shown in Figures S2 (a), (b), and (d), LIPA dominates the composition. However, when LIPA is held constant in Figure S2 (c), the substitutive effects among the remaining components become more apparent.

Reference: McGregor D, Palarea-Albaladejo J, Dall P, et al. Cox regression survival analysis with compositional covariates: Application to modelling mortality risk from 24-h physical activity patterns[J]. Statistical Methods in Medical Research, 2020, 29(5): 1447-1465.

Compositional Data Analysis: Theory and Applications | Wiley [EB/OL]//Wiley.com. [2024-11-24]. https://www.wiley.com/enus/Compositional+Data+Analysis%3A+Theory+and+Applications-p-9780470711354.i

**Appendix S6. The selection of confounders**

**Figure S4. Directed Acyclic Graph (DAG) guiding the selection of observed confounders.**
The DAG illustrates the hypothesized relationships between the exposure (daily movements), the outcome (self-harm), and covariates included in the multivariable models (green nodes). The node in grey ("Unmeasured Confounders") represents latent factors, such as genetic predispositions or specific adverse family life events, which were not available in the dataset but are acknowledged as potential sources of residual confounding.

Literature Review of Potential Confounders

Previous studies have identified several factors associated with self-harm (Fliege et al., 2009). Sociodemographic and educational factors include female sex for self-harm (male for suicide in most countries), low socioeconomic status, LGBT orientation, and limited educational attainment (Hawton et al., 2012). Additionally, residing in low- and middle-income countries (LMICs) is associated with a higher prevalence of self-harm, and adolescents’ body mass index (BMI) has been linked to self-harming behaviors (Kinoshita et al., 2012). Adverse life events and family adversity are also critical factors, encompassing parental separation or divorce, parental death, adverse childhood experiences, history of physical or sexual abuse, parental mental disorders, family history of suicidal behavior, marital or family discord, bullying, and interpersonal difficulties (Johnson et al., 2002). Psychiatric and psychological factors such as mental disorders (especially depression, anxiety, and attention-deficit hyperactivity disorder), BMI, substance misuse, impulsivity, low self-esteem, poor problem-solving skills, perfectionism, and hopelessness further increase self-harm risk (Fliege et al., 2009; Hawton et al., 2012).

Distinct gender differences have been observed in daily physical activity and sedentary behaviors (Nielsen et al., 2011), with socioeconomic status influencing children’s patterns of physical activity (Drenowatz et al., 2010). Parental marital status affects children’s likelihood of engaging in outdoor activities and integrating into peer groups (Kriel et al., 2016; Rodrigues et al., 2018), while interpersonal difficulties can hinder participation in physical activities (Dwyer et al., 2008; Wilk et al., 2018). Obese children exhibit poorer daily movement patterns, with longer sedentary periods and less physical activity compared to their normal-weight peers (Hemmingsson & Ekelund, 2007; Schwarzfischer et al., 2017). Other factors, such as premature birth, daycare attendance, parental BMI, and employment status, influence preschool children’s physical activity patterns (Finn et al., 2002), though evidence linking these factors to self-harm is limited. Studies suggest that physical activity (PA) improves outcomes related to substance misuse, particularly alcohol, among adults (Thompson et al., 2020), though the impact of substance misuse on PA patterns in adolescents remains underexplored. Additionally, PA has been associated with reduced impulsivity (Ghahramani et al., 2016; Leasure & Neighbors, 2014), improved decision-making (Sone et al., 2017), and beneficial mental health outcomes in children and adolescents, particularly by alleviating depressive symptoms and enhancing self-concept (Dale et al., 2019; Ekeland et al., 2005). While studies have examined the association between PA and bullying, it remains unclear if bullying directly affects PA patterns in youth; however, PA may improve coping mechanisms and resilience in bullying situations (Liu et al., 2024; Méndez et al., 2019). Further research has also established associations between adverse childhood experiences (ACEs) and PA, with PA interventions found to bolster resilience in children with ACEs (Lewis-de Los Angeles, 2022; Norris & Norris, 2021). Children with parental mental disorders often experience poorer sleep quality and shorter sleep duration (Roberts et al., 2020). Similarly, children and adolescents with depressive symptoms frequently exhibit disrupted sleep patterns, further influencing their mental health outcomes (Marino et al., 2021).

Selection of Observed Confounders (Green Nodes in DAG)

The selection of confounders was based on existing literature and directed acyclic graph (DAG) analyses. We included variables that are known to be associated with both the exposure and outcome, thereby minimizing potential bias in estimating independent associations. Specifically, age, sex, ethnicity, annual family income, BMI z-score, parents’ marital status, children’s mental health history, children’s interpersonal difficulties, parental mental disorders, and residential area were incorporated as confounders. Given that our cohort is from the United Kingdom, we replaced "country of residence" with "residential area" to reflect within-country variability in social contexts. These factors were chosen due to their documented associations with self-harm and daily movement patterns, ensuring a more robust and accurate model.

Exclusion of Unavailable Confounders (Grey Node in DAG)

We acknowledge that other potential confounders identified in the literature—specifically factors such as sexual orientation (LGBT), substance misuse, bullying involvement, child impulsivity, and detailed family functioning measures—were relevant but not included in the final multivariable models. This exclusion was strictly due to data availability constraints (e.g., lack of data at age 7 or high missingness) in the specific MCS dataset release accessed for this study.

Consequently, to ensure methodological transparency, these factors are classified as "Unmeasured Confounders." As explicitly illustrated in the revised Directed Acyclic Graph (DAG) above, we acknowledge that these unmeasured factors may serve as sources of residual confounding in the association between daily movement behaviors and self-harm.

References

Calzo, J. P., Roberts, A. L., Corliss, H. L., Blood, E. A., Kroshus, E., & Austin, S. B. (2014). Physical activity disparities in heterosexual and sexual minority youth ages 12–22 years old: roles of childhood gender nonconformity and athletic self-esteem. *Annals of Behavioral Medicine*, 47(1), 17-27.

Dale, L. P., Vanderloo, L., Moore, S., & Faulkner, G. (2019). Physical activity and depression, anxiety, and self-esteem in children and youth: An umbrella systematic review. *Mental Health and Physical Activity,* 16, 66-79.

Drenowatz, C., Eisenmann, J. C., Pfeiffer, K. A., Welk, G., Heelan, K., Gentile, D., & Walsh, D. (2010). Influence of socio-economic status on habitual physical activity and sedentary behavior in 8-to 11-year old children. *BMC Public Health*, 10(1), 1-11.

Dwyer, J., Needham, L., Simpson, J. R., & Heeney, E. S. (2008). Parents report intrapersonal, interpersonal, and environmental barriers to supporting healthy eating and physical activity among their preschoolers. *Applied Physiology, Nutrition, and Metabolism,* 33(2), 338-346.

Ekeland, E., Heian, F., Hagen, K. B., Abbott, J., & Nordheim, L. (2005). Exercise to improve self‐esteem in children and young people. *Campbell Systematic Reviews*, 1(1), 1-52.

Finn, K., Johannsen, N., & Specker, B. (2002). Factors associated with physical activity in preschool children. *The Journal of Pediatrics*, 140(1), 81-85.

Fliege, H., Lee, J. R., Grimm, A., & Klapp, B. F. (2009). Risk factors and correlates of deliberate self-harm behavior: A systematic review. *Journal of Psychosomatic Research*, 66(6), 477-493.

Ghahramani, M. H., Sohrabi, M., & Besharat, M. A. (2016). The effects of physical activity on impulse control, attention, decision-making and motor functions in students with high and low impulsivity. *Biosciences Biotechnology Research Asia,* 13(3), 1689-1696.

Hawton, K., Saunders, K. E., & O'Connor, R. C. (2012). Self-harm and suicide in adolescents. *The Lancet,* 379(9834), 2373-2382.

Hemmingsson, E., & Ekelund, U. (2007). Is the association between physical activity and body mass index obesity dependent?. *International Journal of Obesity*, 31(4), 663-668.

Johnson, J. G., Cohen, P., Gould, M. S., Kasen, S., Brown, J., & Brook, J. S. (2002). Childhood adversities, interpersonal difficulties, and risk for suicide attempts during late adolescence and early adulthood. *Archives of General Psychiatry,* 59(8), 741-749.

Kinoshita, K., Kinoshita, Y., Shimodera, S., Nishida, A., Inoue, K., ... & Okazaki, Y. (2012). Not only body weight perception but also body mass index is relevant to suicidal ideation and self-harming behavior in Japanese adolescents. *The Journal of Nervous and Mental Disease*, 200(4), 305-309.

Kriel, C., Schreck, C. M., & Watson, F. (2016). Participation of divorced single parents and their children in outdoor activities to improve attitudes and relationships. *South African Journal for Research in Sport, Physical Education and Recreation*, 38(3), 69-79.

Leasure, J. L., & Neighbors, C. (2014). Impulsivity moderates the association between physical activity and alcohol consumption. *Alcohol,* 48(4), 361-366.

Lewis-de Los Angeles, W. W. (2022). Association between adverse childhood experiences and diet, exercise, and sleep in pre-adolescents. *Academic Pediatrics*, 22(8), 1281-1286.

Liu, Y., Chen, Z., Wang, P., & Xu, L. (2024). Relationship between bullying behaviors and physical activity in children and adolescents: A systematic review and meta-analysis. *Aggression and Violent Behavior*, 101976.

Marino, C., Andrade, B., Campisi, S. C., Wong, M., Zhao, H., Jing, X., ... & Szatmari, P. (2021). Association between disturbed sleep and depression in children and youths: a systematic review and meta-analysis of cohort studies. *JAMA Network Open*, 4(3), e212373.

Méndez, I., Ruiz-Esteban, C., & Ortega, E. (2019). Impact of the physical activity on bullying. *Frontiers in Psychology,* 10, 1520.

Nielsen, G., Pfister, G., & Bo Andersen, L. (2011). Gender differences in the daily physical activities of Danish school children. *European Physical Education Review,* 17(1), 69-90.

Norris, G., & Norris, H. (2021). Building resilience through sport in young people with adverse childhood experiences. *Frontiers in Sports and Active Living*, 3, 663587.

Roberts, C. M., Harper, K. L., Bistricky, S. L., & Short, M. B. (2020). Bedtime behaviors: Parental mental health, parental sleep, parental accommodation, and children’s sleep disturbance. *Children's Health Care*, 49(2), 115-133.

Rodrigues, D., Padez, C., & Machado-Rodrigues, A. M. (2018). Active parents, active children: The importance of parental organized physical activity in children’s extracurricular sport participation. *Journal of Child Health Care,* 22(1), 159-170.

Schwarzfischer, P., Weber, M., Gruszfeld, D., Socha, P., Luque, V., ... & Grote, V. (2017). BMI and recommended levels of physical activity in school children. *BMC Public Health*, 17, 1-9.

Sone, T., Kawachi, Y., Abe, C., Otomo, Y., Sung, Y. W., & Ogawa, S. (2017). Attitude and practice of physical activity and social problem-solving ability among university students. *Environmental Health and Preventive Medicine,* 22, 1-8.

Thompson, T. P., Horrell, J., Taylor, A. H., Wanner, A., Husk, K., Wei, Y., ... & Wallace, G. (2020). Physical activity and the prevention, reduction, and treatment of alcohol and other drug use across the lifespan (The PHASE review): A systematic review. *Mental Health and Physical Activity,* 19, 100360.

Wilk, P., Clark, A. F., Maltby, A., Smith, C., Tucker, P., & Gilliland, J. A. (2018). Examining individual, interpersonal, and environmental influences on children’s physical activity levels. *SSM-Population Health*, 4, 76-85.
